# Evaluation of Oxford Nanopore MinION RNA-Seq Performance for Human Primary Cells

**DOI:** 10.3390/ijms22126317

**Published:** 2021-06-12

**Authors:** Ilaria Massaiu, Paola Songia, Mattia Chiesa, Vincenza Valerio, Donato Moschetta, Valentina Alfieri, Veronika A. Myasoedova, Michael Schmid, Luca Cassetta, Gualtiero I. Colombo, Yuri D’Alessandra, Paolo Poggio

**Affiliations:** 1 Centro Cardiologico Monzino IRCCS, 20131 Milan, Italy; ilaria.massaiu@cardiologicomonzino.it (I.M.); paola.songia@cardiologicomonzino.it (P.S.); mattia.chiesa@cardiologicomonzino.it (M.C.); vincenza.valerio@cardiologicomonzino.it (V.V.); donato.moschetta@cardiologicomonzino.it (D.M.); valentina.alfieri@cardiologicomonzino.it (V.A.); veronika.myasoedova@cardiologicomonzino.it (V.A.M.); gualtiero.colombo@cardiologicomonzino.it (G.I.C.); yuri.dalessandra@cardiologicomonzino.it (Y.D.); 2 Dipartimento di Medicina Clinica e Chirurgia, Università degli Studi di Napoli Federico II, 80131 Napoli, Italy; 3 Dipartimento di Scienze Farmacologiche e Biomolecolari, Università degli Studi di Milano, 20133 Milano, Italy; 4 Genexa AG, Dienerstrasse 7, CH-8004 Zürich, Switzerland; michael.schmid@genexa.ch; 5 The Queen’s Medical Research Council Centre for Reproductive Health, University of Edinburgh, Edinburgh EH16 4TJ, UK; luca.cassetta@ed.ac.uk

**Keywords:** nanopore technology, RNA-seq, MinION, sequencing depth, multiplexed samples, barcoding, SQK-PCS109

## Abstract

Transcript sequencing is a crucial tool for gaining a deep understanding of biological processes in diagnostic and clinical medicine. Given their potential to study novel complex eukaryotic transcriptomes, long-read sequencing technologies are able to overcome some limitations of short-read RNA-Seq approaches. Oxford Nanopore Technologies (ONT) offers the ability to generate long-read sequencing data in real time via portable protein nanopore USB devices. This work aimed to provide the user with the number of reads that should be sequenced, through the ONT MinION platform, to reach the desired accuracy level for a human cell RNA study. We sequenced three cDNA libraries prepared from poly-adenosine RNA of human primary cardiac fibroblasts. Since the runs were comparable, they were combined in a total dataset of 48 million reads. Synthetic datasets with different sizes were generated starting from the total and analyzed in terms of the number of identified genes and their expression levels. As expected, an improved sensitivity was obtained, increasing the sequencing depth, particularly for the non-coding genes. The reliability of expression levels was assayed by (i) comparison with PCR quantifications of selected genes and (ii) by the implementation of a user-friendly multiplexing method in a single run.

## 1. Introduction

Knowledge about the human genome plays a crucial role in modern medicine [1,2]. In-depth analysis of tissue transcriptomes, leveraging the power of genome-wide gene expression investigation, is increasingly used for clinical decisions in the new era of precision medicine [3]. RNA sequencing (RNA-Seq), based on next-generation sequencing technologies, is the current method of choice for quantifying gene expression at the genomic level with higher depth and accuracy than probe-based microarray approaches [4].

Recently, third-generation sequencing (TGS) approaches have been designed to overcome some of the limitations of the second-generation sequencing (SGS) technologies, which rely on short-read length analysis [5]. Despite massive throughput, the use of SGS for de novo transcriptome assembly and analysis of large structural variations remains challenging [6,7]. TGS technologies have the advantage of capturing many full-length single-molecule transcripts, avoiding the problematic errors of assembly processes, and reducing the time-to-results window [8].

The MinION sequencer, released by Oxford Nanopore Technologies (ONT), is a new, low-cost, handheld device that processes thousands of long reads in parallel, using 50 ng starting material [9,10]. The process is based on the passage of DNA/RNA strands through a biological nanopore, generating base-specific changes in electrical conductivity and leading to the identification of specific sequences using a neural network. Although Nanopore long-read sequencing suffers from some limitations compared to short-read methods, such as the high error rate during the base-calling step and the sensitivity to RNA degradation, significant advantages in identifying novel RNA molecules and complex isoforms have been widely demonstrated in previous works [6,11,12,13,14,15]. In particular, the long-read sequencing technology was more efficient in quantifying long non-coding RNAs [16]. Moreover, thanks to its ability to perform rapid long-read sequencing analysis requiring minimal supporting laboratory infrastructure or technical expertise, MinION has been widely used for the diagnosis of viral disease [17,18,19]. It played a crucial role in supporting the COVID-19 pandemic, particularly in isolated or resource-poor settings [20,21,22].

The turnaround time and cost for ONT RNA-Seq can be further reduced by sequencing multiple samples on a single run [19,23,24,25]. Wick et al. [23] sequenced 12 bacterial DNA isolates simultaneously on a single MinION flow cell using the ONT native barcoding kit. The long reads were then combined in a hybrid assembly with Illumina data to fully resolve the bacterial genome. Recently, King et al. [24] proposed a rapid workflow for multiplexed sequencing of influenza A viruses using the ONT technology for real-time analysis, in combination with a one-step RT-PCR and the Rapid Barcoding Kit.

As with any RNA-Seq study, the read depth is one of the most important factors to reach the desired level of accuracy and sensitivity, in addition to the number of biological replicates. Indeed, the read depth changes in accordance with the purpose of the study, requiring a significant increase when low-expression genes are to be evaluated. Numerous investigations regarding the performance of short-read RNA-Seq with varying reads depth have been published [26,27,28,29,30]. Conversely, a very limited number of studies focusing on the real capabilities of the MinION sequencing platform are available [6,15,31,32,33,34,35]. In particular, an RNA-Seq evaluation with the latest PCR-cDNA sequencing kit (i.e., SQK-PCS109) is missing. This kit is highly recommended for users who have a limited amount of input material, want to optimize their sequencing experiment for throughput, would like to identify and quantify full-length transcripts, and are interested in differential gene expression.

Here, we have qualitatively and quantitatively analyzed the performance of MinION through the sequencing of human primary cardiac fibroblasts, both in terms of the number of detected genes and corresponding quantifications, by sequentially changing the number of reads, employing the SQK-PCS109 kit. Our goal was to estimate, according to the target accuracy of a given study, a suitable sequencing depth to obtain a reliable number of detected genes as well as their expression levels from human primary cells.

## 2. Results

**Figure 1:**
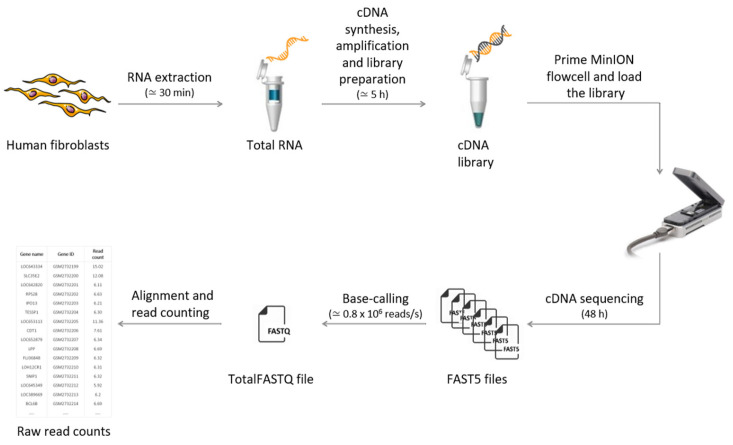
Study workflow. RNA was extracted from human cardiac fibroblast cells. Then, cDNA library preparation (including size, quantity, and quality analysis), base calling, read mapping, and counting were performed.

The workflow diagram of the study is reported in Figure 1.

### 2.1. Sequencing Performance Changing the Number of Reads

**Figure 2:**
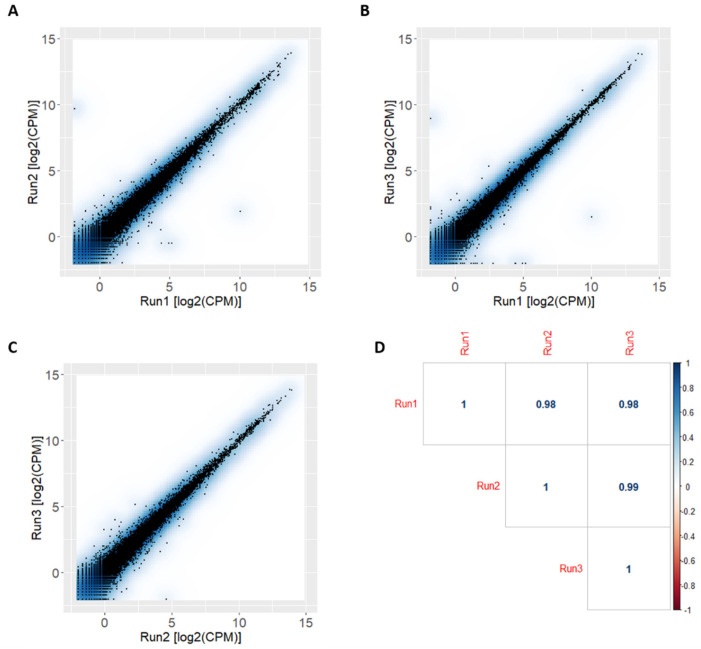
Correlation analysis comparing the three libraries. (**A**) Scatter plot of log_2_(CPM) values from run1 vs. run2. (**B**) Scatter plot of log_2_(CPM) values from run1 vs. run3. (**C**) Scatter plot of log_2_(CPM) values quantified from run2 vs. run3. (**D**) Correlation matrix with Pearson correlation coefficients.

Three cDNA libraries, prepared from poly-adenosine (poly-A) RNA of three human primary cardiac fibroblast samples, were sequenced by an ONT MinION sequencer using R9.4 flow cells. The comparison of the gene expression levels, expressed as log_2_(CPM), showed a strong correlation among these three independent replicates (Pearson’s correlation coefficient (r_p_) equal to 0.98; Figure 2).

Thus, data obtained from these runs were combined to obtain a large dataset (hereinafter DS100) for the evaluation of ONT performance as a function of read number. DS100 was composed of more than 48 million reads (about 23 gigabases), with an average length of 483.2 base pairs (bp) that passed the quality filter. We used this total dataset to generate progressively fractional synthetic subsets (90% to 5%, i.e., DS90 to DS5). The total and mapped reads are reported in Table 1 for each dataset.

**Figure 3:**
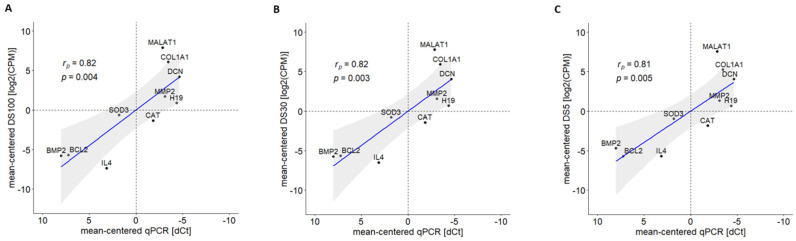
Correlation analysis of gene expression levels obtained from qRT-PCR and ONT sequencing using (**A**) DS100, (**B**) DS30, and (**C**) DS5. Interleukin 4 (IL4), metastasis associated lung adenocarcinoma transcript 1 (MALAT1), collagen type I alpha 1 chain (COL1A1), decorin (DCN), matrix metallopeptidase 2 (MMP2), h19 imprinted maternally expressed transcript (H19), catalase (CAT), superoxide dismutase 3 (SOD3), BCL2 apoptosis regulator (BCL2), and bone morpho-genetic protein 2 (BMP2) genes were selected, and qPCR experiments were performed. Pearson correlation coefficients (r_p_) between log_2_(CPM) and dCt were computed and reported with the respective *p*-value (*p*).

We obtained similar length distributions of reads among the different subsets (Supplementary Figure S1 and Supplementary Table S1). Moreover, a strong agreement was found between the average gene expression levels obtained from the eleven datasets (DS100–DS5), even when comparing the two extreme datasets, DS5 and DS100 (r_p_ = 0.97 and *p* < 10^−5^; Supplementary Figure S2). The reliability of the results was evaluated by comparing the quantifications obtained from DS5, DS30, and DS100 with the dCt values of ten selected genes (IL4, MALAT1, COL1A1, DCN, MMP2, H19, CAT, SOD3, BCL2, and BMP2) detected by qPCR. There was a significant correlation between each dataset quantification and the qPCR values (r_p_ ≥ 0.8 and *p* < 0.01; Figure 3). Of note, the DS30 subset (about 14 million reads) is equivalent to a single flow-cell run. Indeed, the SQK-PCS109 kit can generate 10–15 million reads in 48 h per flow cell.

**Figure 4:**
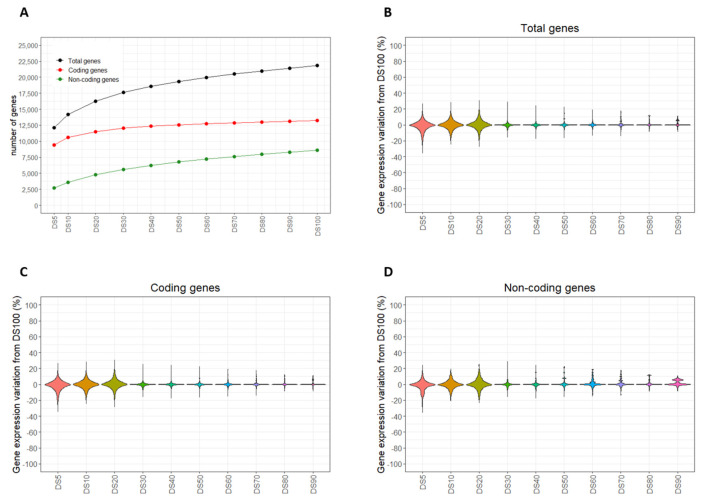
Sequencing results of total, coding, and non-coding genes as a function of the read depth. (**A**) Number of total, coding, and non-coding genes in each dataset (black, red, and green lines, respectively). Distributions of gene expression variation of (**B**) total, (**C**) coding, and (**D**) non-coding genes in each subset.

The sequencing performance, when changing the number of reads, was assessed by evaluating the number of detected genes as well as the corresponding quantification levels based on different read depths. Moreover, we investigated coding and non-coding genes considering the Ensembl 97 database (GRCh38.p12). A total of 21,816 expressed genes were detected using DS100, and the detection sensitivity decreased after reducing the size of the subsets (from DS90 to DS5; Figure 4A, black line, and Supplementary Table S2). In particular, we identified 17,633 genes in DS30 and 12,114 in DS5. The detection trend revealed a significant improvement from DS5 up to DS30. Indeed, the number of expressed genes detected by DS30 was about 50% higher than that for DS5. Instead, compared with DS30, an increase of about 25% was reached using the largest dataset, DS100. As expected, the genes with low expression levels were challenging to detect (Supplementary Figure S3).

Focusing on the biotype classification, we observed that >60% of the genes were annotated as protein-coding (Figure 4A, red line, and Supplementary Table S2) and the remaining part was composed of non-coding genes (Figure 4A, green line, and Supplementary Table S2). The improvement in the detection of coding genes reached a plateau after DS20. In particular, from DS5 to DS20, we observed an increment of about 22% in the number of coding genes. As for the non-coding genes, an increment of >100% was achieved in DS30 compared with DS5, and 53% going from DS30 to DS100.

The gene quantifications obtained from each subset (from DS90 to DS5) were compared to the results of DS100 (as reference) to compute the gene expression variation (%GEV). A substantial reduction in the %GEV was shown considering the subsets larger than DS20 (Figure 4B and Supplementary Table S3). The %GEV values obtained for coding and non-coding genes had similar characteristics (Figure 4C,D; Supplementary Tables S4 and S5). However, in the latter, we observed a slightly higher %GEV.

Finally, the %GEV values at different ranges were investigated in the two biotypes. In particular, coding and non-coding genes were grouped into high, mid-high, mid-low, and low expression subsets and analyzed. An overall negative correlation between the %GEV and the expression class was observed for all the subsets (Supplementary Figures S4 and S5).

### 2.2. Multiplex Sequencing

**Figure 5:**
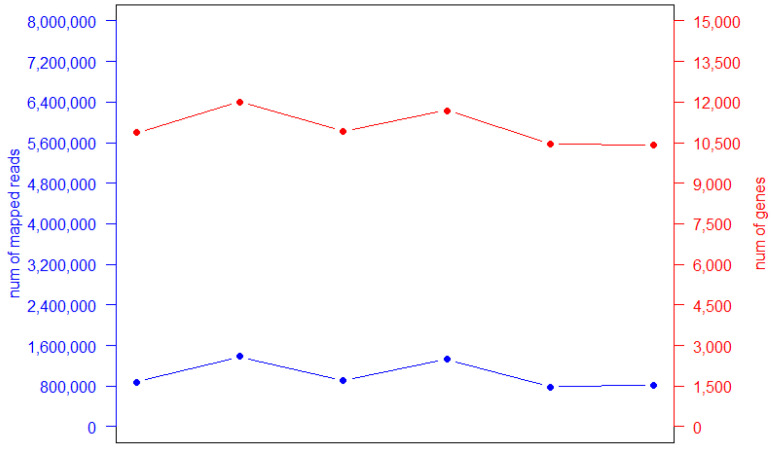
Sequencing results of each barcoded sample. Number of mapped reads (**red**) and identified genes (**blue**) obtained for each barcoded sample.

To further validate the results obtained from the synthetic subsets, we sequenced one sample with six different barcodes in a single run and compared it to DS5 (about two million reads). More than 10 million reads passed the quality filter (99.9% of the overall passed reads). These reads were then assigned to each barcode and six different datasets were generated. An average of 1.7 million high-quality reads made up each dataset, of which about 60% were mapped to the reference genome, and more than 10,000 genes were identified in each barcoded sample (5.26% CV; Table 2 and Figure 5), similar to the DS5 synthetic subset.

**Figure 6:**
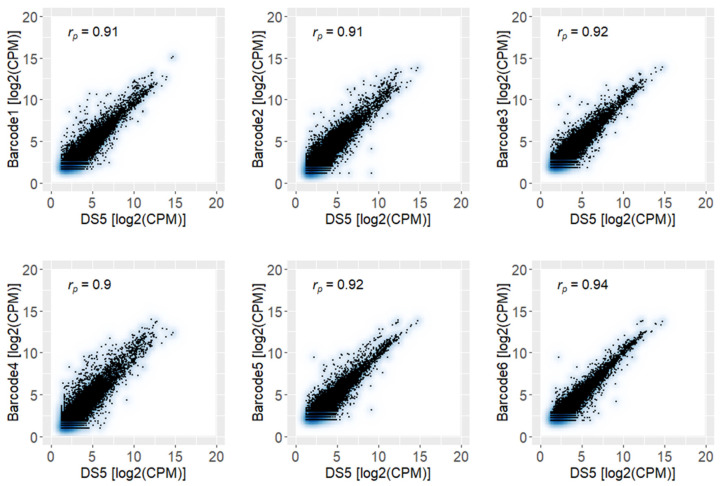
Correlation analysis of results obtained from the six barcoded samples with the smallest subset DS5.

Furthermore, the expression levels of the barcoded samples were compared to the values quantified in the DS5 subset (Figure 6). The high correlation (r_p_ ≥ 0.9 and *p* < 10^−5^) between each barcoded sample and the DS5 subset revealed the reproducibility and high quality of the obtained results.

## 3. Discussion

The ability to sequence long DNA fragments provided by the ONT MinION system offers easier assembly of complex genomes than short-read methods do, but its high error rate is still an obstacle [6,7]. Thanks to the decreased turnaround time at a low cost compared to short-read sequencing, and the continuous performance improvements, also including the option to sequence multiple samples in a single run [19,23,24], this pocket-sized device is widely used both for scientific research and clinical applications [36]. Different transcriptomic studies exploited the ability of this technology to uncover the diversity of alternative splicing isoforms and their expression levels [12,14,15]. Using MinION sequencing, Bolisetty et al. [37] identified over 7000 isoforms for Dscam1, the most complicated alternatively spliced gene known in nature, with an average identity of full-length alignments > 90%, by MinION sequencing. Moreover, thanks to its long-read capability, de novo sequencing of microbial, viral, and eukaryotic whole genomes is more easily obtainable [18,38,39]. For these reasons, MinION has proven to be a valuable support for the ongoing worldwide pandemic caused by severe acute respiratory syndrome coronavirus 2 (SARS-CoV-2) [20]. Its capability to perform rapid long-read sequencing analysis, with flexible scalability and accurate consensus-level sequence determination [21], offered key knowledge of virus transmission and evolution as well as for vaccine development [22].

Focusing on RNA studies, to date, ONT sequencing has achieved relevant results in terms of uncovered transcripts, quantification of expression levels, and differential expression analysis, comparable with Illumina technology [6,40]. One of the most important factors for the proper design of RNA-Seq experiments is the sequencing depth. This parameter represents the number of reads collected during each run for a given sample, and in general, its increment leads to an improvement of the sequencing results. However, a unique optimal number of required reads cannot be claimed, and a high depth is normally required to study novel or less abundant transcripts. Nonetheless, a higher depth of sequencing inevitably involves an increment in costs.

Identification of the optimal read depth in function of the aim of the experiments and the complexity of the target transcriptome is a crucial aspect [41]. Indeed, several studies on RNA-Seq performance that involved changing the depth were carried out for short-read technologies [26,27,28,29,30]. Conversely, few references only for the earlier versions of ONT technology are present in the literature, leaving the experimental design somewhat sketchy [6,15,34,35]. Furthermore, these studies were not focused on the evaluation of the optimal read depth, but they were performed to compare this new technology with Illumina platforms. Thus, using the latest available PCR-cDNA kit (i.e., SQK-PCS109), we assessed the ability of the ONT MinION sequencing platform to identify and accurately quantify annotated genes by changing the number of reads, in human primary cells.

With our approach (i.e., poly-A RNA-Seq), more than 12,000 genes were detected with about two million reads, where about 22% belonged to the non-coding biotype. Signs of saturation were obtained at about 14 million reads, representing the typical amount of data achieved in a single run. Similar outcomes were achieved in terms of quantifications, where all the evaluated synthetic datasets had a comparable gene expression. Taking into account the biotype classification, we confirmed that an accurate sequencing of non-coding genes is particularly challenging, since they are typically expressed in low levels [42].

In conclusion, our study is the first, to the best of our knowledge, to show how many genes can be accurately identified and quantified as a function of sequencing depth employing the latest PCR-cDNA ONT kit. We intended to provide new users approaching ONT RNA-Seq a guideline on the optimal read depth to be reached, obtaining a good compromise between accuracy of results, costs, and processing time.

That being said, our study has some limitations to be pointed out. The aim of our study was to analyze the performance of MinION RNA-Seq when varying the number of reads; thus, we did not compare our results with any short-read technologies. Additionally, our data were generated employing human primary cardiac fibroblasts; thus, the number of genes related to the identified depth may not be applicable to other cell types.

## 4. Materials and Methods

### 4.1. Sample and Library Preparation

Valve interstitial cells (VICs) were isolated from three human stenotic valves, and RNA was extracted using the Total RNA Purification Plus Kit (Norgen Biotek, Thorold, ON, Canada), according to the manufacturer’s instructions. We pooled samples and prepared three cDNA libraries following the recommendations of the Nanopore cDNA-Seq protocol for the SQK-PCS109 kit. Briefly, we employed RT primers to convert only poly-adenylated RNA into cDNA. For the multiplex run, we used six different ad hoc designed barcoded sequences (Supplementary Table S6). cDNA synthesis was performed using 50 ng of total RNA per sample. RT and strand-switching primers were provided by ONT with the SQK-PCS109 kit. Following RT, PCR amplification was performed using the LongAmp Taq 2X Master Mix (New England Biolabs, Ipswich, MA, USA) and the following cycling conditions: 1 cycle (95 °C for 30 s), 18 cycles (95 °C for 15 s, 62 °C for 15 s, and 65 °C for 3 min), and 1 last cycle (65 °C for 15 min). PCR products were purified using Agencourt AMPure XP beads (Beckman Coulter, Brea, CA, USA). The cDNA sequencing libraries were prepared using a total of 200 fmol of cDNA each.

### 4.2. MinION Sequencing

Nanopore libraries were sequenced using a MinION Mk1B sequencing device with R9.4 flow cells. Sequencing was controlled and data were generated using ONT MinKNOW software (v3.4.12). Runs were terminated after 48 h and FAST5 files were generated.

### 4.3. Data Processing

DNA bases were called from FAST5 files using ONT Guppy GPU (v3.4.5) in high accuracy mode [43]. Reads with an average Phred quality score, which measures the confidence based on the estimated error rate, lower than 7 were discarded. The first three runs were combined in a single dataset, named DS100, and used as the reference for studying the sequencing performance. Then, 10 additional datasets were generated by randomly sampling 90% (DS90), 80% (DS80), 70% (DS70), 60% (DS60), 50% (DS50), 40% (DS40), 30% (DS30), 20% (DS20), 10% (DS10), and 5% (DS5) of DS100. To assess the reliability of the sampling, this procedure was implemented 10 times for each dataset.

### 4.4. Bioinformatic Analysis

Reads were aligned to the 22 diploid chromosomes of the GRCh38 human genome reference with minimap2 (v2.1, default parameters except for -ax splice) [44]. SAM-to-BAM format conversion as well as an assessment of the alignment quality were performed using Samtools (v 1.10) [45]. The FeatureCounts software (v2.0.0) [46], included in the Subread package, was used to count the mapped reads. Finally, the expression of each gene was reported as counts per million transformed in logarithmic scale (log_2_(CPM)) [47]. For the multiplex run, the quality-checked reads were de-multiplexed and trimmed for barcodes using the Cutadapt function (v1.15) [48], before the alignment and counting procedures. Genes with a read count greater than 3 were deemed as expressed.

### 4.5. Performance Evaluation

Computational analyses were implemented using the R software environment (v 3.6.0) [49]. Pearson’s correlation coefficients (r_p_) were computed to check the reproducibility of the quantifications. The average number of genes detected and the coefficient of variation (CV) between the replicates across each subset were computed to evaluate the dispersion generated by subsetting. Correlations between each of the subsets and with the entire dataset were assessed. The biotype was assigned based on the Ensembl 97 database (GRCh38.p12). Genes were grouped into protein-coding and non-coding, the latter including long non-coding RNA (lncRNA), non-coding RNA (ncRNA), and pseudogenes, following the Ensembl Genome Browser annotation (https://www.ensembl.org/info/genome/genebuild/biotypes.html, accessed date 28 January 2021).

To evaluate the results as a function of read depth, we compared the number and expression level of genes detected among each subset with respect to DS100. These analyses were repeated for coding and non-coding genes separately.

The gene expression variations (%GEV) of the log_2_(CPM) values obtained in the subsets (from DS90 to DS5) were compared to that calculated in the total dataset (DS100) as follows:

%GEV_DSi,j_ = (log_2_(CPM_TOT,j_) − log_2_(CPM_DSi,j_)/log_2_(CPM_TOT,j_)) × 100 (1)

where %GEV_DSi,j_ is the gene expression variation (expressed as a percentage) relative to the i-th subset (from DS90 to DS5) and the j-th gene, log_2_(CPM_TOT,j_) is the expression value obtained with the total dataset (DS100) for the j-th gene, and log_2_(CPM_DSi,j_) is the expression value using the i-th subset (from DS90 to DS5) for the j-th gene. These values were computed only for the genes identified at least from the 80% of replicates of the i-th subset.

The variation distribution was depicted by violin plots. In addition, we repeated the same analysis after dividing the detected coding and non-coding genes into quartiles, which represented high (Q4), medium-high (Q3), medium-low (Q2), and low expression (Q1) transcripts.

### 4.6. Quantitative Real-Time PCR

We tested the robustness of our results correlating gene expression levels obtained from the DS100, DS50, and DS5 datasets with values detected by qPCR, performed on an ABI Prism 7900 HT (Applied Biosystems, Foster City, CA, USA) with SYBR Green dye (New England BioLabs, Ipswich, MA, USA), according to the manufacturers’ instructions. To consider expression levels spread over a broad range, we selected glyceraldehyde-3-phosphate dehydrogenase (GAPDH) as the reference gene and metastasis-associated lung adenocarcinoma transcript 1 (MALAT1), collagen type I alpha 1 chain (COL1A1), decorin (DCN), matrix metallopeptidase 2 (MMP2), H19 imprinted maternally expressed transcript (H19), catalase (CAT), superoxide dismutase 3 (SOD3), BCL2 apoptosis regulator (BCL2), bone morphogenetic protein 2 (BMP2), and interleukin 4 (IL4) as the target genes to be evaluated (Supplementary Table S7). For each gene, the cycle threshold (Ct) value was determined and the dCt value was calculated (target Ct–GAPDH Ct).

## Figures and Tables

**Table 1:** The numbers of total and mapped reads are reported for each subset (DS90–DS5) and the total dataset (DS100). The numbers of mapped reads for the subsets are the average among 10 randomly picked replicated subsets with the same size (CV < 0.8%).

Dataset	Avg. Total Reads	Avg. Mapped Reads
DS100	48,495,343	28,239,507
DS90	43,645,809	25,415,207
DS80	38,796,274	22,592,768
DS70	33,946,740	19,768,060
DS60	29,097,206	16,943,277
DS50	24,247,672	14,098,354
DS40	19,398,137	11,302,374
DS30	14,548,603	8,461,139
DS20	9,699,069	5,368,370
DS10	4,849,534	2,676,107
DS5	2,424,767	1,340,245

**Table 2:** Sequencing data of each barcoded sample.

Dataset	Total Reads	Mapped Reads	Number of Total Genes
Barcode1	1,293,001	878,970	10,869
Barcode2	2,029,051	1,382,710	12,006
Barcode3	1,401,956	909,050	10,917
Barcode4	2,640,182	1,331,922	11,690
Barcode5	1,871,851	784,207	11,442
Barcode6	1,196,100	821,091	10,418

## Data Availability

The data presented in this study and R code are openly available in Zenodoo at doi:10.5281/zenodo.4767610.

## Supplementary Materials

The following are available online at https://www.mdpi.com/article/10.3390/ijms22126317/s1. Table S1: Average length and the coefficient of variation (CV) among the 10 replicates are reported for each dataset. Table S2: Number of total, coding and, non-coding genes identified from each dataset. Table S3: Quantile values of gene expression variations of each subset respect to DS100 considering total genes. Table S4: Quantile values of gene expression variations of each subset respect to DS100 considering coding genes. Table S5: Quantile values of gene expression variations of each subset respect to DS100 considering non-coding genes. Table S6: Barcoded sequences, Table S7: qPCR primers. Figure S1: Length distributions of sequenced reads composing (A) the total dataset DS100 and (B) each replicate of DS5 (as an example). Figure S2: Correlation analysis of the gene expression values obtained from the different datasets. Figure S3: Variation of gene detection among the eleven datasets (DS5-DS100), in terms of percentage, in function of the average expression levels (log2(CPM)). Figure S4: Distributions of gene expression variation for coding genes with high, mid-high, mid-low, and low expression. Figure S5: Distributions of gene expression variation for non-coding genes with high, mid-high, mid-low, and low expression.
