# Supplementary material for: Evaluation of Oxford Nanopore MinION RNA-Seq Performance for Human Primary Cells
Source: Int J Mol Sci. 2021 Jun 12;22(12):6317. doi: 10.3390/ijms22126317 (PMC8231517; doi:10.3390/ijms22126317)

Evaluation of Oxford Nanopore MinION RNA-seq performance for human primary cells.

Ilaria Massaiu [1], Paola Songia [1], Mattia Chiesa [1], Vincenza Valerio [1,2], Donato Moschetta [1,3], Valentina Alfieri [1], Veronika A. Myasoedova [1], Michael Schmid [4], Luca Cassetta [5], Gualtiero I. Colombo [1], Yuri D'Alessandra [1,†] and Paolo Poggio [1,*†]

[1] Centro Cardiologico Monzino IRCCS, Milan, Italy; ilaria.massaiu@cardiologicomonzino.it (I.M); paola.songia@cardiologicomonzino.it (P.S); mattia.chiesa@cardiologicomonzino.it (M.C); valentina.alfieri@cardiologicomonzino.it (V.A); veronika.myasoedova@cardiologicomonzino.it (V.A.M); gualtiero.colombo@cardiologicomonzino.it (G.I.C); yuri.dalessandra@cardiologicomonzino.it (D.Y)
[2] Università degli Studi di Napoli Federico II, Dipartimento di Medicina Clinica e Chirurgia, Napoli, Italy; vincenza.valerio@cardiologicomonzino.it (V.V)
[3] Università degli Studi di Milano, Dipartimento di Scienze Farmacologiche e Biomolecolari, Milano, Italy; donato.moschetta@cardiologicomonzino.it (D.M)
[4] Genexa AG, Dienerstrasse 7, CH-8004, Zürich, Switzerland; michael.schmid@genexa.ch (M.S)
[5] The Queen's Medical Research Council Centre for Reproductive Health, University of Edinburgh, Edinburgh, UK; luca.cassetta@ed.ac.uk (L.C)
[*] Correspondence: paolo.poggio@cardiologicomonzino.it
[†] These authors contributed equally.

Supplementary Table S1. Average length and the coefficient of variation (CV) among the 10 replicates are reported for each dataset.

DATASET	AVERAGE LENGTH	CV %
DS100	483.2	--
DS90	483.2	0.009
DS80	483.1	0.02
DS70	483.1	0.02
DS60	483.2	0.03
DS50	483.2	0.04
DS40	483.2	0.05
DS30	483.1	0.04
DS20	491.7	0.04
DS10	491.5	0.12
DS5	491.7	0.17

Supplementary Table S2. Number of total, coding and, non-coding genes identified from each dataset. The respective average values and coefficient of variation (CV) among 10 replicates are reported.

Dataset	Number of total genes		Number of coding genes		Number of non-coding genes	
	Average	CV (%)	Average	CV (%)	Average	CV (%)
DS100	21,816	--	13,242	--	8,574	--
DS90	21,424	0.06	13,139	0.04	8,245	0.15
DS80	20,993	0.09	13,023	0.07	7,970	0.25
DS70	20,503	0.17	12,897	0.15	7,606	0.34
DS60	19,945	0.19	12,744	0.15	7,200	0.43
DS50	19,329	0.29	12,560	0.17	6,769	0.68
DS40	18,583	0.16	12,338	0.20	6,245	0.48
DS30	17,633	0.15	12,030	0.15	5,603	0.29
DS20	16,251	0.21	11,499	0.16	4,752	0.52
DS10	14,172	0.23	10,590	0.20	3,579	0.67
DS5	12,114	0.26	9,419	0.29	2,695	0.74

Supplementary Table S3. Quantile values of gene expression variations of each subset respect to DS100 considering total genes. The first quartile, median and third quartile of the distribution of gene expression variation for the total genes.

Dataset	Q1	Median	Q3
DS90	-0.17	0.026	0.88
DS80	-0.26	-0.001	0.31
DS70	-0.42	0.002	0.49
DS60	-0.51	-0.016	0.39
DS50	-0.62	-0.007	0.48
DS40	-0.78	-0.044	0.41
DS30	-0.98	-0.076	0.43
DS20	-2.54	0.17	3.18
DS10	-2.81	-0.13	2.62
DS5	-4.26	-0.64	2.07

Supplementary Table S4. Quantile values of gene expression variations of each subset respect to DS100 considering coding genes. The first quartile, median and third quartile of the distribution of gene expression variation for the protein coding genes.

Dataset	Q1	Median	Q3
DS90	-0.19	0.001	0.25
DS80	-0.28	-0.008	0.23
DS70	-0.42	-0.01	0.34
DS60	-0.57	-0.013	0.48
DS50	-0.64	-0.025	0.41
DS40	-0.75	-0.056	0.40
DS30	-0.98	-0.096	0.43
DS20	-2.5	0.21	3.16
DS10	-2.82	-0.061	2.63
DS5	-4.3	-0.64	2.17

Supplementary Table S5. Quantile values of gene expression variations of each subset respect to DS100 considering non-coding genes. The first quartile, median and third quartile of the distribution of gene expression variation for the protein coding genes.

Dataset	Q1	Median	Q3
DS90	-0.11	0.21	5.44
DS80	-0.17	0.013	1.22
DS70	-0.40	0.040	1.58
DS60	-0.48	0.044	2.76
DS50	-0.55	0.015	1.03
DS40	-0.91	-0.013	0.48
DS30	-0.98	-0.026	0.44
DS20	-2.67	-0.061	3.36
DS10	-2.81	-0.33	2.41
DS5	-4.13	-0.62	1.63

Supplementary Table S6. Barcoded sequences.

Name	Sequence
Barcode1	5phos/ ACTTGCCTGTCGCTCTATCTTC TAATCCACTCCGAA TTTTTTTTTTTTTTTTTTTTVN
Barcode2	5phos/ ACTTGCCTGTCGCTCTATCTTC GGCCTTCTTGTCAC TTTTTTTTTTTTTTTTTTTTVN
Barcode3	5phos/ ACTTGCCTGTCGCTCTATCTTC ATTAGGTGTGGCGG TTTTTTTTTTTTTTTTTTTTVN
Barcode4	5phos/ ACTTGCCTGTCGCTCTATCTTC CGGAAGAAGAAGAA TTTTTTTTTTTTTTTTTTTTVN
Barcode5	5phos/ ACTTGCCTGTCGCTCTATCTTC AGGATATGAGACGA TTTTTTTTTTTTTTTTTTTTVN
Barcode6	5phos/ ACTTGCCTGTCGCTCTATCTTC TCGTCTTGGTAGTA TTTTTTTTTTTTTTTTTTTTVN

Supplementary Table S7. qPCR primers.

Gene	Direction	Sequence (5′ – 3′)
GAPDH	Forward	ACA TCG CTC AGA CAC CAT G
	Reverse	TGT AGT TGA GGT CAA TGA AGG G
MALAT1	Forward	CAC CGA AGG CTT AAA GTA GGA C
	Reverse	GCT GAC ACT TCT CTT GAC CTT AG
COL1A1	Forward	CCC CTG GAA AGA ATG GAG ATG
	Reverse	TCC AAA CCA CTG AAA CCT CTG
DCN	Forward	CTC AGC TAT TTC TTC TAC CTC TCC
	Reverse	CAT CAG TTC TCT AGA CCA GTC AG
MMP2	Forward	TCC ACC ACC TAC AAC TTT GAG
	Reverse	GTG CAG CTG TCA TAG GAT GT
H19	Forward	CTT TAC AAC CAC TGC ACT ACC T
	Reverse	GCT GTT CCG ATG GTG TCT T
CAT	Forward	ACC GAG AGA GAA TTC CTG AGA
	Reverse	GCC TTG GAG TAT TTG GTA ATG TC
SOD3	Forward	TGC AGC TCT CTT TTC AGG AG
	Reverse	GCA GGC AGG AAC ACA GTA G
IL4	Forward	CAG TTC TAC AGC CAC CAT GAG
	Reverse	GTT TCA GGA ATC GGA TCA GC
BCL2	Forward	GAT GAC TGA GTA CCT GAA CCG
	Reverse	AGC CAG GAG AAA TCA AAC AGA G

BMP2	Forward<br>Reverse	TTT GAC CAG AGT TTT TCC ATG TG<br>GAA GCA GCA ACG CTA GAA GA

Supplementary Figure S1. Length distributions of sequenced reads composing (A) the total dataset DS100 and (B) each replicate of DS5 (as an example).

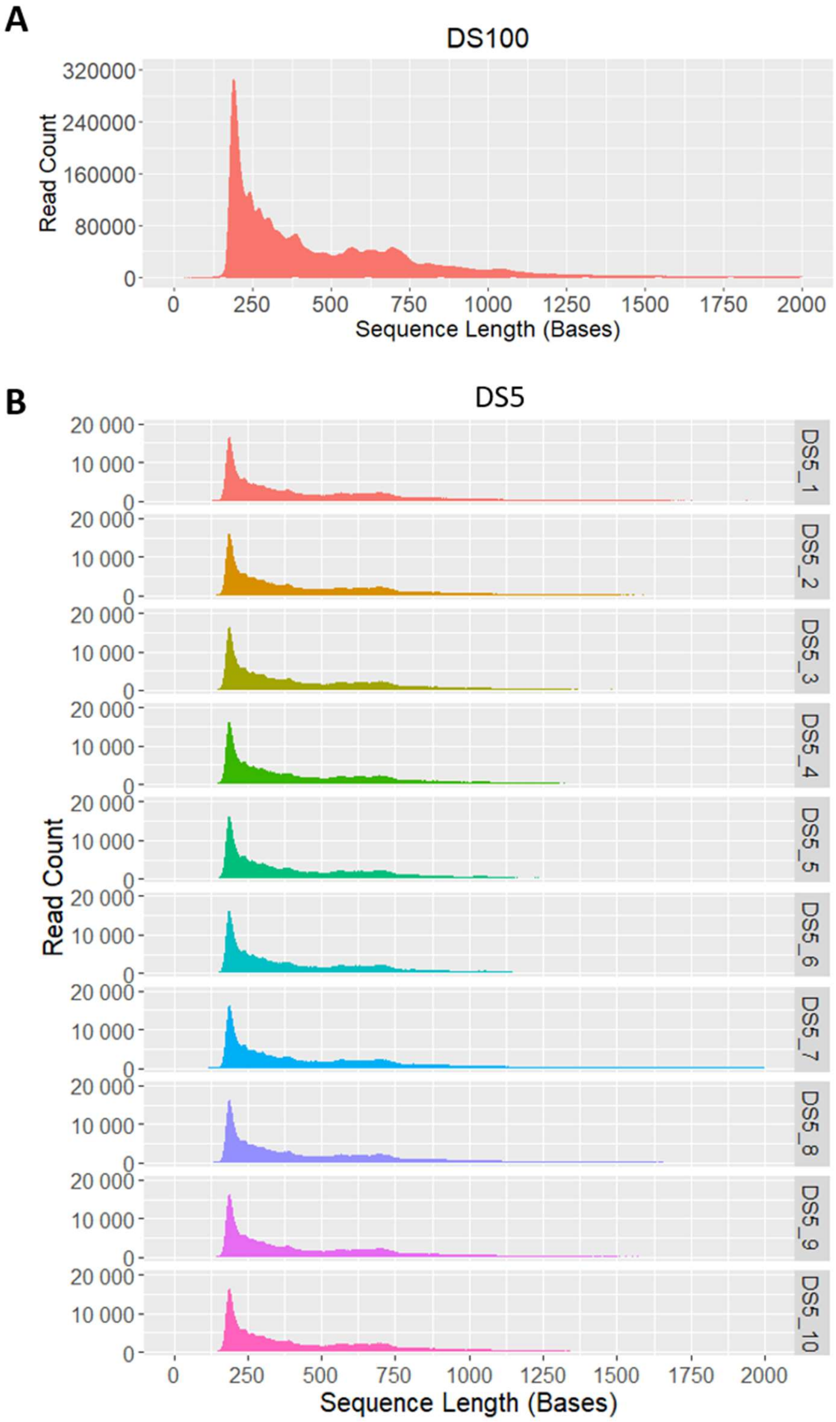

Supplementary Figure S2. Correlation analysis of the gene expression values obtained from the different datasets. (A) Scatter plot of log2(CPM) values from DS100 vs. DS30. (B) Scatter plot of log2(CPM) values from DS100 vs. DS5. (C) Scatter plot of log2(CPM) values from DS30 versus DS5. Pearson correlation coefficient (rp) between log2(CPM) was computed and reported with the respective p-value (p). (D) Correlation matrix with each rp of the eleven datasets (DS100-DS5).

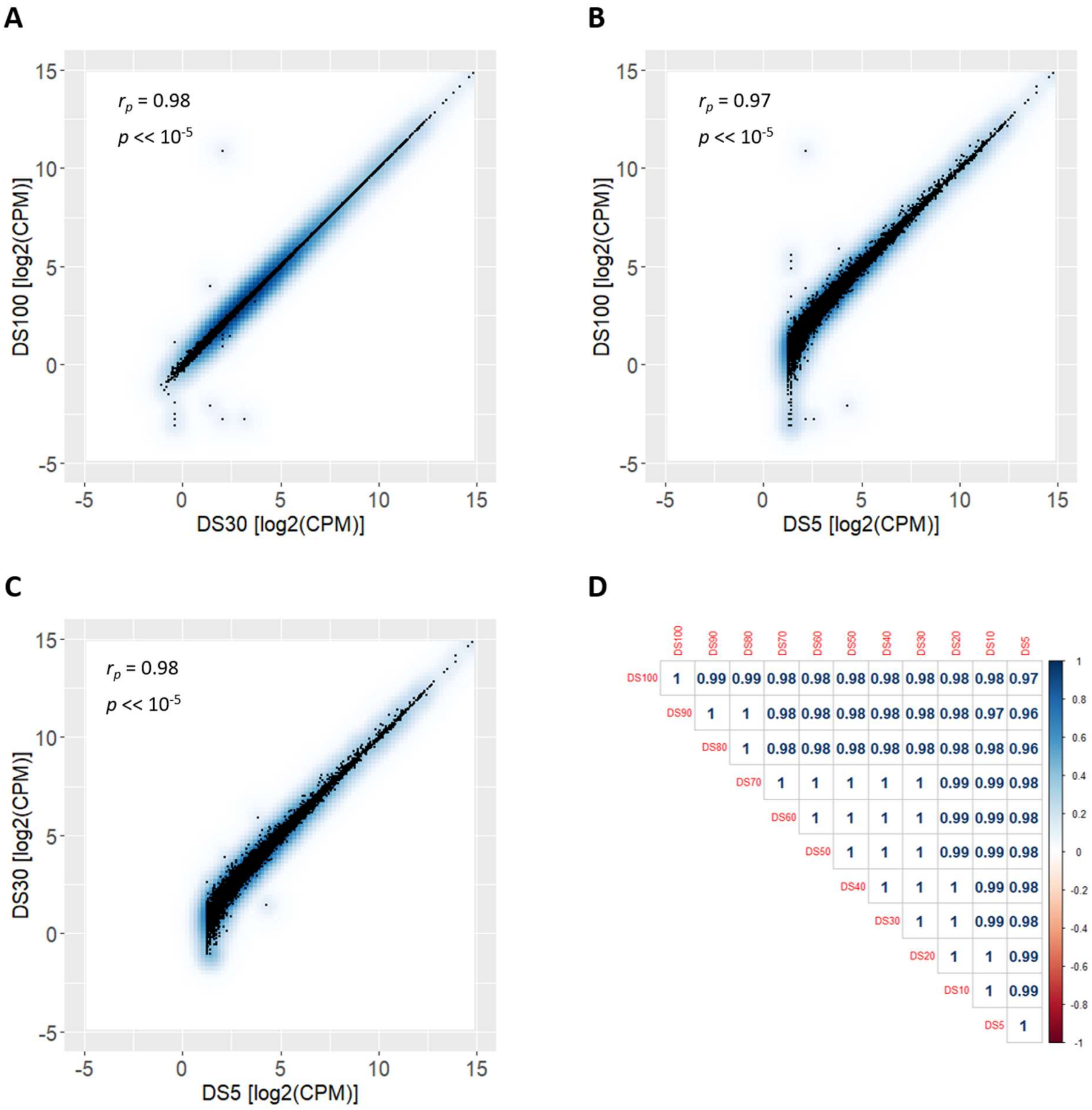

Supplementary Figure S3. Variation of gene detection among the eleven datasets (DS5-DS100), in terms of percentage, in function of the average expression levels (log_2(CPM)).

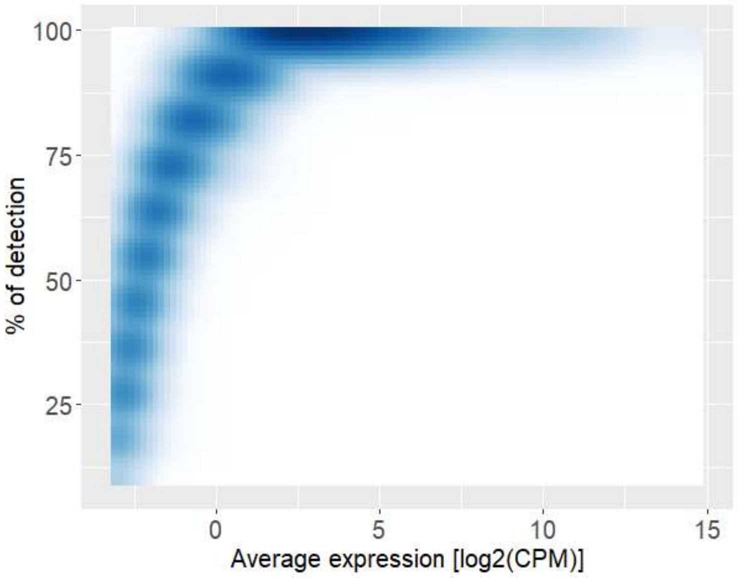

Supplementary Figure S4. Distributions of gene expression variation for coding genes with high, mid-high, mid-low, and low expression. The percentages of gene expression variation (%GEV) of each subset (DS5-DS90) from the total dataset (DS100) were represented as violin plots. (A) Coding genes with expression higher than Q3. (B) Coding genes with an expression between Q3 and the median. (C) Coding genes with an expression between the median value and Q1. (D) Coding genes with an expression lower than Q1.

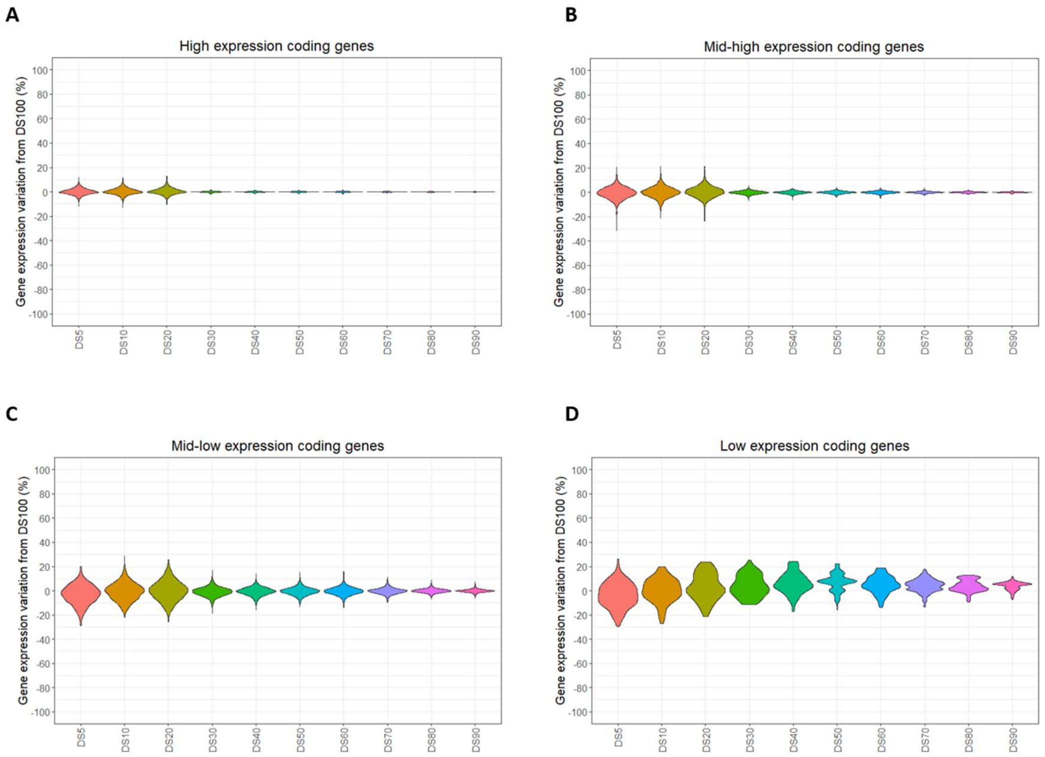

Supplementary Figure S5. Distributions of gene expression variation for non-coding genes with high, mid-high, mid-low, and low expression. The percentages of gene expression variation (%GEV) of each subset (DS5-DS90) from the total dataset (DS100) were represented as violin plots. (A) Non-coding genes with expression higher than Q3. (B) Non-coding genes with an expression between Q3 and the median. (C) Non-coding genes with an expression between the median value and Q1. (D) Non-coding genes with an expression lower than Q1.

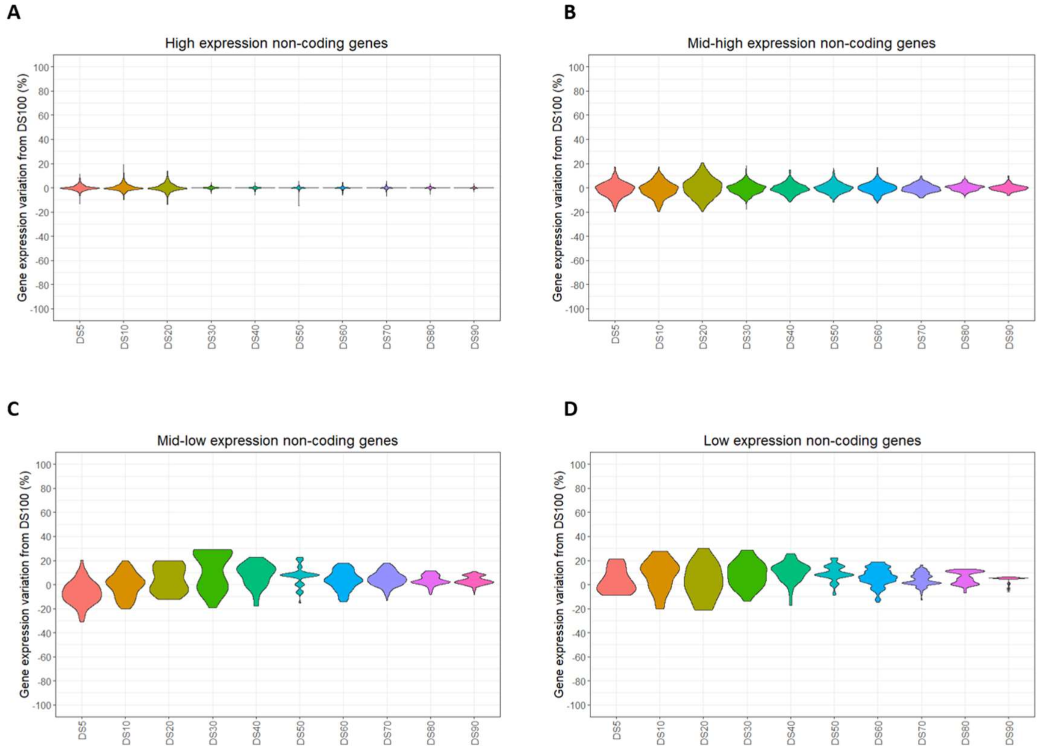

Supplement: Supplementary file 1 [file ijms-22-06317-s001.zip › ijms-1210554-supplementary.pdf]
